# Potential Cross-Reactive Immunity to COVID-19 Infection in Individuals With Laboratory-Confirmed MERS-CoV Infection: A National Retrospective Cohort Study From Saudi Arabia

**DOI:** 10.3389/fimmu.2021.727989

**Published:** 2021-09-17

**Authors:** Anas A. Khan, Ahmed A. Alahmari, Yasir Almuzaini, Fahad Alamri, Yousef Mohammad Alsofayan, Alhanouf Aburas, Saleh Al-Muhsen, Maria Van Kerkhove, Saber Yezli, Gregory R. Ciottone, Abdullah M. Assiri, Hani A. Jokhdar

**Affiliations:** ^1^Department of Emergency Medicine, College of Medicine, King Saud University, Riyadh, Saudi Arabia; ^2^Global Center of Mass Gatherings Medicine, Ministry of Health, Riyadh, Saudi Arabia; ^3^Immunology Research Laboratory, Department of Pediatrics, College of Medicine, King Saud University Medical City, King Saud University, Riyadh, Saudi Arabia; ^4^Infectious Hazards Management, Health Emergencies Programme, World Health Organization, Geneva, Switzerland; ^5^Department of Emergency Medicine, Harvard Medical School, Boston, MA, United States; ^6^Deputyship of Public Health, Ministry of Health, Riyadh, Saudi Arabia

**Keywords:** coronavirus disease 2019, Middle East respiratory syndrome coronavirus, Saudi Arabia, cross-immunity, mortality

## Abstract

**Background:**

A growing number of experiments have suggested potential cross-reactive immunity between severe acute respiratory syndrome coronavirus-2 (SARS-CoV-2) and previous human coronaviruses. We conducted the present retrospective cohort study to investigate the relationship between previous Middle East respiratory syndrome-coronavirus (MERS-CoV) infection and the risk of SARS-CoV-2 infection as well as the relationship between previous MERS-CoV and COVID-19-related hospitalization and mortality.

**Methods:**

Starting in March 2020, we prospectively followed two groups of individuals who tested negative for COVID-19 infection. The first group had a previously confirmed MERS-CoV infection, which was compared to a control group of MERS-negative individuals. The studied cohort was then followed until November 2020 to track evidence of contracting COVID-19 infection.

**Findings:**

A total of 82 (24%) MERS-positive and 260 (31%) MERS-negative individuals had COVID-19 infection. Patients in the MERS-positive group had a lower risk of COVID-19 infection than those in the MERS-negative group (Risk ratio [RR] 0.696, 95% confidence interval [CI] 0.522-0.929; p =0.014). The risk of COVID-19-related hospitalization in the MERS-positive group was significantly higher (RR 4.036, 95% CI 1.705-9.555; p =0.002). The case fatality rate (CFR) from COVID-19 was 4.9% in the MERS-positive group and 1.2% in the MERS-negative group (p =0.038). The MERS-positive group had a higher risk of death than the MERS-negative group (RR 6.222, 95% CI 1.342-28.839; p =0.019). However, the risk of mortality was similar between the two groups when death was adjusted for age (p =0.068) and age and sex (p =0.057). After controlling for all the independent variables, only healthcare worker occupation and >1 comorbidity were independent predictors of SARS-CoV-2 infection.

**Interpretation:**

Individuals with previous MERS-CoV infection can exhibit a cross-reactive immune response to SARS-CoV-2 infection. Our study demonstrated that patients with MERS-CoV infection had higher risks of COVID-19-related hospitalization and death than MERS-negative individuals.

## Introduction

Coronaviruses (CoVs) within the *Coronaviridae* family are a group of enveloped, positive-strand RNA viruses that infect numerous animal species (1). However, since the severe acute respiratory syndrome (SARS) outbreak in 2003, it was realized that CoVs could cause devastating zoonotic diseases that raise concerns regarding the significant health threats of these viral strains in humans (2). Six species of human CoV are known, of which three, SARS-CoV, Middle East respiratory syndrome-coronavirus (MERS-CoV), and SARS-CoV-2, are highly pathogenic and cause pneumonia and systemic symptoms in humans (3, 4). These strains share many similarities, such as genomic structure, route of transmission, and clinical manifestations (5). Furthermore, they have similar sequence homology and antigenic epitopes that can induce an adaptive immune response (6). However, unlike SARS-CoV-2, SARS-CoV and MERS-CoV displayed only limited person-to-person spread, leading to fewer confirmed cases (7).

The MERS outbreak originated in Saudi Arabia in 2012, where the virus was mainly transmitted through dromedary camels (8). As of December 2020, a total of 2566 laboratory-confirmed cases and 882 deaths due to MERS were reported globally, leading to a high fatality rate of 34% (9). The clinical features of MERS-CoV infection vary substantially and can range from asymptomatic or flu-like symptoms to severe pneumonia, acute respiratory distress, multiorgan failure, and death (10). To date, there are no approved vaccines or specific therapies for MERS-CoV infection (8).

On the other hand, coronavirus disease 2019 (COVID-19), caused by SARS-CoV-2 which was first reported in China in December 2019, is an ongoing global pandemic that has affected more than 110 million persons to date (11). The global COVID-19 fatality rate is approaching 2.2%, and it notably rises to 49% in critically ill patients (11, 12). The COVID-19 pandemic poses a significant challenge to healthcare services and societies globally. The pandemic has also exerted a tremendous socioeconomic toll, the consequences of which are yet to be recognized. A significant contributor to the difficulty of adequately managing the outbreak is the sheer volume of cases, which threatens overwhelming the available resources (such as ventilators and ICU beds) of healthcare facilities (13). Thus, a global effort was cumulated to develop a number of effective vaccines. Nonetheless, several unresolved issues remain concerning the longevity of these vaccines and their efficacy against emerging viral variants (14).

A growing number of research studies have suggested potential cross-reactive immunity between SARS-CoV-2 and previous human CoVs, which is thought to stem from the high sequence similarity between these viruses, leading to reactive CD4+ T cells (6, 15–17). A case series by Barry et al. (18), assessed the clinical characteristics of 99 hospitalized patients with COVID-19 in a MERS-CoV center in the Kingdom of Saudi Arabia (KSA) and reported no co-occurrence of MERS-CoV among SARS-CoV-2-infected persons. Immunity resulting from previous MERS-CoV infection has been suggested to underlie the lower mortality rates of COVID-19 (16, 19). These results accord with Kim et al. (20), who found persistence of antibodies against spike protein in 70 patients with previous MERS-CoV for three years after the infection (21). A more recent report detected MERS-CoV–specific neutralizing antibodies for six years post-infection in 48 patients with previous MERS-CoV infection. A case report of a 31-year-old physician with COVID-19 and previous MERS-CoV infection suggested that prior MERS-CoV infection provided partial immunity leading to mild disease (22).

The current statistics reveal that nearly 85% of the total MERS-CoV cases worldwide occurred in the KSA. As of December 2020, there were 2167 laboratory-confirmed MERS cases in the KSA and 804 related deaths (9). On the other hand, a total of 362,979 COVID-19 cases were reported in the KSA by the end of December 2020 (23). Thus, the KSA provides a unique setting where both MERS-CoV and SARS-CoV-2 are circulating. Hence, we conducted the present retrospective cohort study to investigate the relationship between previous MERS-CoV infection and the risk of SARS-CoV-2 infection, as well as the relationship between previous MERS-CoV and COVID-19-related hospitalization and mortality.

## Materials and Methods

The Institutional Review Board of the Ministry of Health (MoH) in Saudi Arabia approved the study protocol (Central/RB log No: 21 -28M). All study procedures were compliant with the principles of the Declaration of Helsinki (24) and local regulatory laws. As the present study was a retrospective chart review, the need for informed consent was waived by the IRB committee. The present manuscript was prepared in compliance with the recommendations of the Strengthening the Reporting of Observational Studies in Epidemiology (STROBE) statement (25).

### Study Design and Population

The present retrospective cohort study retrieved the data of all individuals who were screened for COVID-19 infection in March 2020 in Saudi Arabia. A total of six million individuals were screened from March to September 2020. We included all cases with a previously confirmed MERS-CoV infection who tested negative for SARS-CoV-2 infection during the screening phase and had no previous history of SARS-CoV-2 infection (n =342). In addition, we utilized a random sampling technique with a ratio of 1:3 to include MERS-CoV-negative individuals from the screened population who were negative for SARS-CoV-2 infection at that time. The studied cohort was then followed until December 2020 to track the evidence of contracting SARS-CoV-2 infection. The diagnosis of COVID-19 infection was confirmed only by reverse transcription-polymerase chain reaction (RT-PCR) laboratory tests (26). The data were collected from the Health Electronic Surveillance Network (HESN) database of the Saudi MoH.

### Sample Size Calculation

The calculated sample size for this study was based on the hypothesis that participants with previous MERS-CoV infection might be at least two times less at risk of SARS-CoV-2 infection *versus* the general population. This assumption was based on a pilot test that randomly compared the risk of SARS-CoV-2 infection between 20 cases with previous MERS-CoV infection and a similar number of MERS-CoV-negative individuals. The latest data from Saudi MoH’s official website suggest that the general population has a positivity rate of approximately 10%. Therefore, the minimum sample size given by Fleiss without continuity correction would be approximately 1380 subjects, including 345 previously infected with MERS and 1035 from the general population. This hypothesis assumes a two-sided type 1 error of 5% and a power of 80% (27).

### Data Collection

The data of eligible participants were obtained from the HSEN. Extracted data included demographic characteristics (age, gender, nationality, region, occupation), smoking status, comorbidities that were defined according to the International Statistical Classification of Diseases and Related Health Problems-10 (ICD-10) (28), history of flu vaccine, presence of laboratory-confirmed MERS-CoV infection, the occurrence of COVID-19 infection, COVID-19 symptoms, the severity of symptoms, need for hospitalization, ICU admission, and death. The data underwent thorough data management steps to ensure accuracy and validity. Each variable was checked for any typing or entry mistakes and bizarre or irrelevant observations.

### Study’s Outcomes

The main parameter of the present study was to compare the incidence of SARS-CoV-2 infection between previously confirmed MERS-CoV cases and MERS-CoV-negative individuals. The secondary parameters included the risk of COVID-19-related hospitalization and mortality among previously confirmed MERS-CoV cases.

### Statistical Analysis

Continuous variables are expressed as medians with interquartile ranges (IQRs), and categorical variables are expressed as percentages (%). Categorical variables were analyzed using the chi-square test or Fisher’s exact test. The relative risk and its associated 95% confidence interval were calculated based on MERS-CoV exposure (Yes/No) and the risk of different outcomes, such as SARS-CoV-2 infection, hospitalization, and death. Multivariable logistic regression was used to test the association of important baseline characteristics with the binary outcome of SARS-CoV-2 infection. The following variables were included in the multivariate analysis: age, previous MERS-CoV infection, flu vaccination during the past year, being a healthcare worker, and > 1 comorbidity. Graphical presentations of some important variables were generated using Microsoft Office Excel 2013 for Windows (Microsoft Corporation, USA). All mean and median values, as well as their measures of variability, were formatted to one decimal place. All percentages were rounded to one decimal place. The significance level was two-sided with a type 1 error of 5%. The analysis was performed using Statistical Package for Social Sciences version 24 (IBM SPSS Statistics for Windows, Version 24.0. Armonk, NY: IBM Corp).

## Results

**Figure 1:**
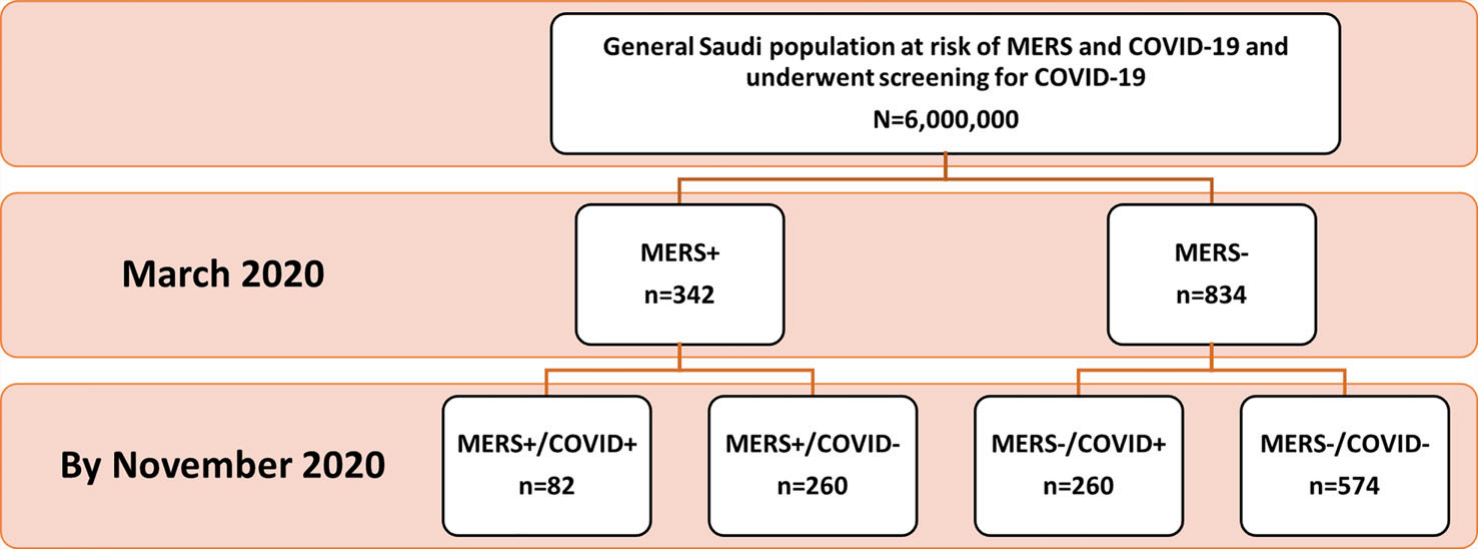
Patient Disposition Flowchart. Out of 6,000,000 people who underwent the national screening on March 2020, 342 patients with a previously confirmed MERS-CoV infection and did not test positive for SARS-CoV-2 infection were identified. The random sample of the control group was taken approximately to be 3 times that of MERS exposed group based on the sample size. Patients were followed-up retrospectively until November 2020 to identify patients who captured SARS-CoV-2 infection.

The MERS-infected population was part of the general Saudi population screened for recent SARS-CoV-2 infection. All MERS-positive cases were selected (n=342) who were negative for SARS-CoV-2 infection at the time of initial screening in March 2020. In addition, a random sample of MERS-negative cases (n=1035) was selected from the screened population; out of them, the complete dataset was retrieved for only 834 individuals. All selected individuals were negative for SARS-CoV-2 infection at that time. The cohort was followed up to November 2020 for evidence of COVID-19 infection. A total of 82 (24%) MERS-CoV-positive and 260 (31%) MERS-CoV-negative individuals had SARS-CoV-2 infection (**Figure 1**).

### Baseline Characteristics of the Study Population

The median age of the studied cohort was 36 (IQR =21) years. The MERS-CoV-positive patients were significantly older than the MERS-CoV-negative patients (p <0.001). The majority of cases in both groups were males (64.4% and 63.7%, respectively; p =0.773). The median duration from MERS-CoV infection and SARS-CoV-2 infection was 3.4 (IQR =3.6) years. Most of the identified cases were of Saudi nationality, with no significant difference between MERS-CoV-positive and MERS-CoV-negative cases (p =0.834). Notably, the number of healthcare workers was significantly higher in the MERS-CoV-positive group than in the MERS-CoV-negative group (34.3% *versus* 12.6%, respectively; p <0.001). MERS-CoV-positive patients were more likely to have one or more comorbidities than MERS-CoV-negative patients (p <0.001). The prevalence of hypertension (p <0.001), diabetes (p <0.001), cardiac diseases (p =0.006), and end-stage renal diseases (p <0.001) was significantly higher in the MERS-CoV-positive patients. Additionally, the flu vaccine was taken in a higher percentage in the MERS-CoV-positive group (41.3%) than in the MERS-CoV-negative group (27%; p =0.012) (**Table 1**).

**Table 1:** Baseline characteristics of the cohort screened for COVID-19.

Characteristic	Total N=1176	MERS+N=342	MERS-N=834	P-value
**Age**				
** Median (IQR)**	36(21)	47(25)	32(17)	<0.001*
0-9	58(4.9%)	3(0.9%)	55(6.6%)	<0.001*
10-19	63(5.4%)	4(1.2%)	59(7.1%)	
20-29	227(19.3%)	24(7.0%)	203(24.3%)	
30-39	339(28.8%)	82(24.0%)	257(30.8%)	
40-49	198(16.8%)	70(20.5%)	128(15.3%)	
50-59	146(12.4%)	73(21.3%)	73(8.8%)	
60-69	89(7.6%)	56(16.4%)	33(4.0%)	
70-79	27(2.3%)	14(4.1%)	13(1.6%)	
≥80	29(2.5%)	16(4.7%)	13(1.6%)	
**Male**	757(64.4%)	218(63.7%)	539(64.6%)	0.773
**Duration since MERS-CoV infection, Median (IQR)**	—	3.4 (3.6)	—	—
**Nationality (n =1162)**				
Saudi	822(70.6%)	243(71.1%)	579(70.4%)	0.834
Non-Saudi	342(29.4%)	99(28.9%)	243(29.6%)	
**Healthcare Worker (n =523)**	88(16.8%)	35(34.3%)	53(12.6%)	<0.001*
**Smoking Status (n =477)**	91(19.1%)	14(19.4%)	77(19.0%)	0.931
**Comorbidities (n =484)**	**484**	**76**	**408**	
One comorbidity	132(27.3%)	47(61.8%)	85(20.8%)	<0.001*
>1 comorbidity	46(9.5%)	22(28.9%)	24(5.9%)	<0.001*
Diabetes	60(12.4%)	28(36.8%)	32(7.8%)	<0.001*
Hypertension	52(10.7%)	27(35.5%)	25(6.1%)	<0.001*
Cardiac	11(2.3%)	5(6.6%)	6(1.5%)	0.006*
Asthma & COPD	19(3.9%)	3(3.9%)	16(3.9%)	0.992
ESRD	9(1.9%)	6(7.9%)	3(0.7%)	<0.001*
Cancer	6(1.2%)	2(2.6%)	4(1.0%)	0.232
**Flu vaccine (n =475)**	139(29.3%)	31(41.3%)	108(27.0%)	0.012*

Bold values represent the total number of patients with co-morbidities. *Statistically significant at p-value <0.05.

**Figure 2:**
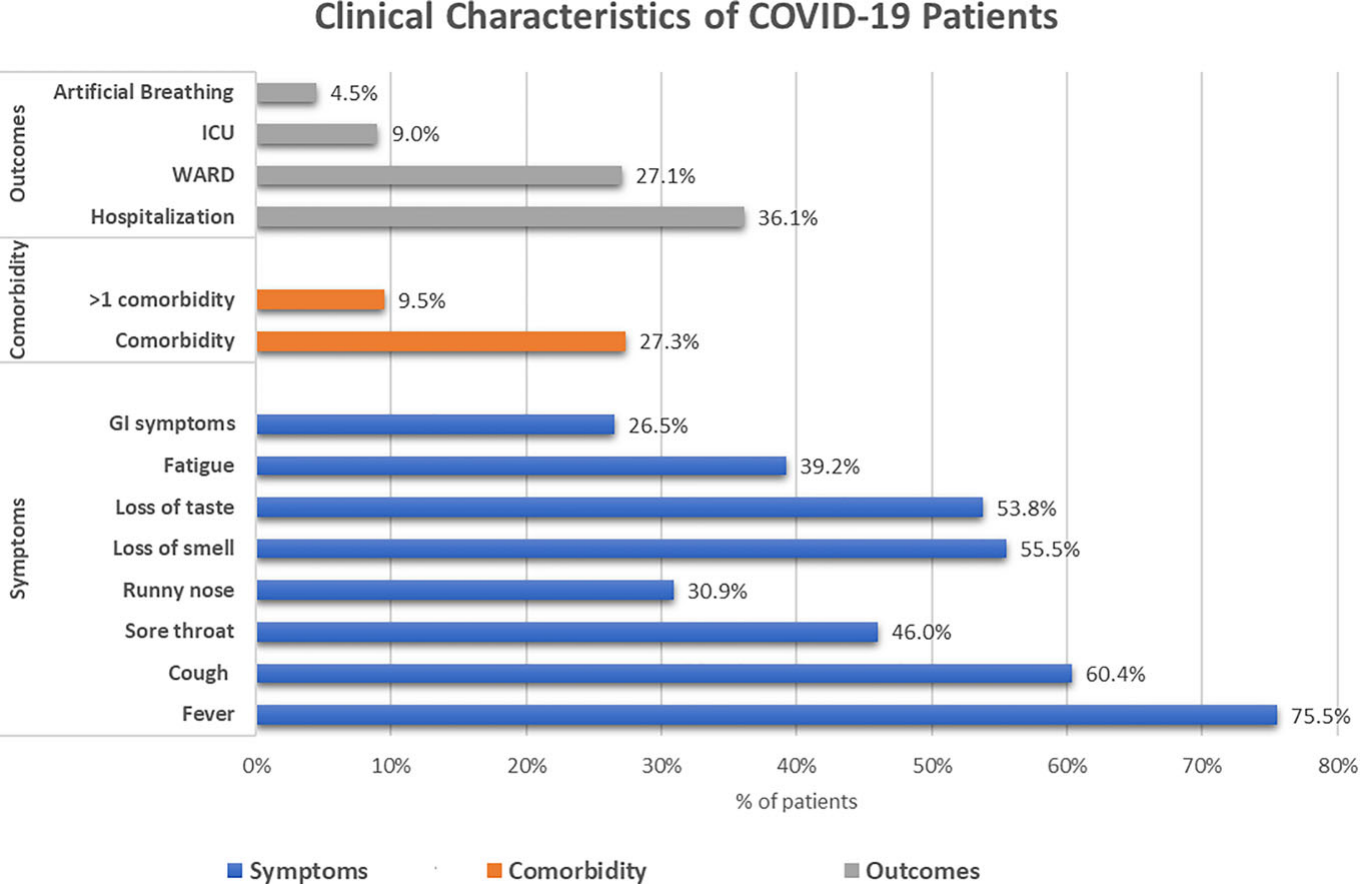
Clinical characteristics of the COVID-19-infected subgroup within the cohort. The blue bars represent the symptomatic presentations of the patients, the orange bars represent the presence of comorbidities, and the grey bars represent the outcomes of the patients.

The COVID-19 subpopulation had a high prevalence of comorbidities (27.3%). Those having more than one comorbidity were (9.5%). Symptoms occurred in 86.5% of the cases, with fever reported by 75.5%, cough by 60.4%, and sore throat by 46% of cases. Regarding the clinical outcomes, hospitalization and ICU admission occurred in 36.1% and 9% of the cases, respectively. The total percentage of cases requiring mechanical or assisted ventilation was 4.5%. The median and IQR of the duration of hospitalization was 7 (16) days (**Figure 2**).

### Risk of SARS-CoV-2 Infection

A total of 342 (29.1%) included cases had SARS-CoV-2 infection by November 2020. The incidence of SARS-CoV-2 infection was significantly higher in the MERS-CoV-negative group (n=260, 31.2%) than in the MERS-CoV-positive group (n=82, 24%; p=0.014) (**Table 2**). Patients in the MERS-CoV-positive group had a significantly lower risk of SARS-CoV-2 infection than those in the MERS-CoV-negative group (RR 0.696, 95% CI 0.522-0.929; p =0.014) (**Table 3**).

**Table 2:** SARS-CoV-2 infection and mortality measures within the cohort subjects.

	Total N= 1176	MERS+N =342	MERS-N=834	P-value
Number of COVID-19 cases	342(29.1%)	82(24.0%)	260(31.2%)	0.014*
Death from COVID-19	7(0.6%)	4(1.2%)	3(0.4%)	0.114
Death from all Causes	14(1.2%)	6(1.8%)	8(1.0%)	0.254
COVID-19 Case Fatality Rate	2.0%	4.9%	1.2%	0.038*

*Statistically significant at p-value <0.05.

**Table 3:** Risk of the MERS-exposed group relative to SARS-CoV-2 infection, symptom presence, hospitalization and death.

	N	RR	95% Confidence Interval	P-value
SARS-CoV-2 infection	342	0.696	0.522-0.929	0.014*
Presence of COVID-19 symptoms	259	0.945	0.404-2.211	0.896
Hospitalization	133	4.036	1.705-9.555	0.002*
Death	245	6.222	1.342-28.839	0.019*
Adjusted Death^#^	245	4.290	0.897-20.511	0.068
Adjusted Death^*^	245	4.651	0.956-22.618	0.057

^#^adjusted for age; *adjusted for age and gender.

### Risk of COVID-19-Related Hospitalization and Death

The risk of having symptoms of COVID-19 was similar between the MERS-CoV-positive and MERS-CoV-negative groups (RR 0.945, 95% CI 0.404-2.211; p =0.89). On the other hand, the risk of COVID-19-related hospitalization in the MERS-CoV-positive group was significantly higher (RR 4.036, 95% CI 1.705-9.555; p =0.002) (**Table 3**).

**Figure 3:**
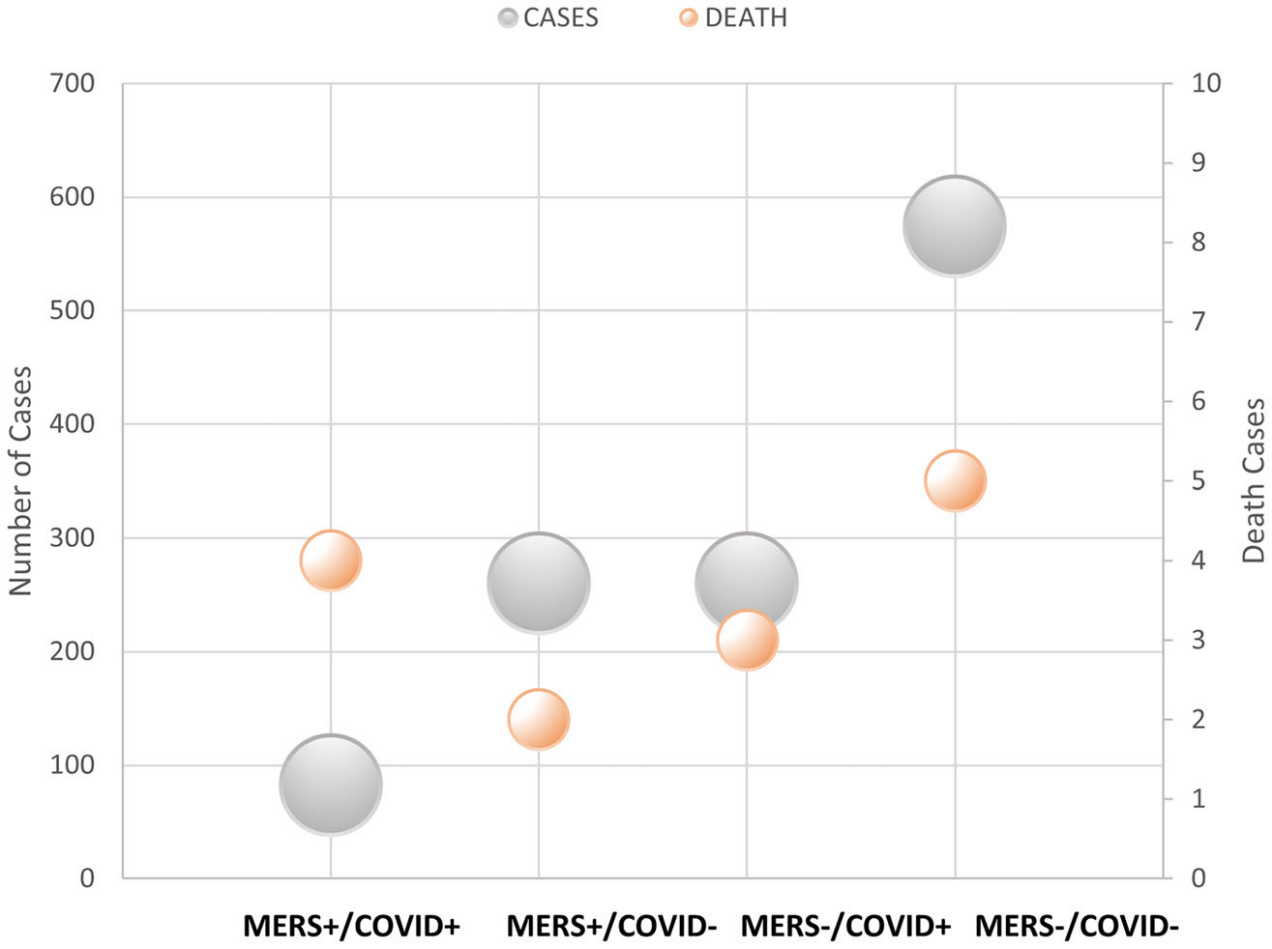
Number of cases and deaths based on MERS and COVID-19 infection.

The number of deaths from COVID-19 was numerically higher in the MERS-CoV-positive group than in the MERS-CoV-negative group (1.2% *versus* 0.4%, respectively; p =0.114). However, the CFR from COVID-19 was 4.9% in the MERS-CoV-positive group and 1.2% in the MERS-CoV-negative group (p =0.038) (**Table 2**). The MERS-CoV-positive group had a higher risk of death than the MERS-CoV-negative group (RR 6.222, 95% CI 1.342-28.839; p =0.019). However, the risk of mortality was similar between the two groups when death was adjusted for age (RR 4.29, 95% CI 0.897-20.511; p =0.068) and age and sex (RR 4.65, 95% CI 0.956-22.62; p =0.057) (**Table 3**). Further stratification of the groups based simultaneously on the presence of the exposure (MERS infection) and presence of the outcome (COVID-19 infection) showed that those who did not contract SARS-CoV-2 infection had approximately the same mortality rate of 0.77% for MERS+/COVID- and 0.87% for MERS-/COVID- (**Figure 3**).

### Independent Predictors of COVID-19 Infection

Multivariable logistic regression was performed to test the association between SARS-CoV-2 infection as a dependent variable and selected independent variables based on their significance in a univariable model or their clinical importance. The model included age in years, previous MERS-CoV infection, flu vaccination, occupation, and >1 comorbidity. After controlling for all the independent variables, only healthcare worker occupation and >1 comorbidity were independently significant, with RRs (95% CI) of 2.336 (1.199-4.550) and 0.389 (0.168-0.898), respectively (**Table 4**).

**Table 4:** Multivariable logistic regression for independent predictors of SARS-CoV-2 infection in the study cohort.

	RR	95% Confidence Interval	P-value
Age in years	1.004	0.991-1.017	0.531
MERS infection	1.181	0.621-2.245	0.612
Flu-vaccine	0.640	0.391-1.049	0.077
Healthcare worker	2.336	1.199-4.550	0.013*
>1 comorbidity	0.389	0.168-0.898	0.027*

*Statistically significant at p-value <0.05.

## Discussion

Since 2002, the world has witnessed the emergence of three human CoV outbreaks. Nonetheless, there is much ambiguity concerning the human immune response to CoVs and whether there is cross-reactive immunity between different human CoVs. To our knowledge, this is the first clinical study that assessed the risk of SARS-CoV-2 infection among individuals with a history of laboratory-confirmed MERS-CoV infection as well as the impact of previous MERS-CoV infection on the clinical course of COVID-19. The outcomes of this study shape the currently ongoing hypotheses regarding the presence of cross-reactive immunity between MERS-CoV and SARS-CoV-2 infection (5). Our results indicated that individuals with MERS-CoV infection had a lower risk of SARS-CoV-2 infection than MERS-negative individuals. Interestingly, patients with MERS-CoV infection had higher risks of COVID-19-related hospitalization and death than MERS-negative individuals. However, the mortality risk of death was similar between MERS-CoV-positive and MERS-CoV-negative cases when death was adjusted for age and sex. In the multivariate analysis, only being a healthcare worker and having >1 comorbidity were independent predictors of COVID-19 infection.

The immune system remains the ideal defense supporting the human’s natural ability to defend itself against foreign pathogens. Innate immunity is the first line of defense against viral infections, including human CoVs. In the context of shared sequence homology of human CoVs (29), it is expected that the innate immune response against SARS-CoV-2 is similar to that of other human CoVs and involves signaling through the Toll-like receptor (TLR) pathway to induce type I and type III interferons (IFNs). Other pro-inflammatory cytokines, such as tumor necrosis factor-β (TNF-β), interleukin-1 (IL-1), and IL-6, are also released (30). This nonspecific antiviral defense potentiates a more specific adaptive immune response through activation of T lymphocytes when the viral antigen is expressed by antigen-presenting cells such as macrophages and dendritic cells (31, 32). T lymphocyte activation produces inflammatory mediators such as interferon-I (INF-I), tumor necrosis factor-β (TNF-β), interleukin-1 (IL-1), IL-6, and monocyte chemoattractant protein-1 known as CCL2. In addition to perforin and granzyme B, a process also occurs in other respiratory infections (33, 34). Animal models for SARS-CoV-1 have suggested that T cells are protective. In mouse models, depletion of CD4^+^ delayed the clearance of the virus and worsened the disease; similarly, T cell augmentation led to rapid clearance of the virus and ameliorated the disease (35, 36). T cell memory is long-lived, and SARS-CoV-1 T cell specificity was identified four years after infection (37, 38). For SARS-CoV-2, T cells have been identified in asymptomatic cases or with mild COVID19 symptoms (39); moreover, the specific T cells of SARS-CoV-2 were detected in contacts of infected cases (40). T cells were fewer in patients with SARS-CoV-2 than in healthy individuals (41). Humoral immunity is essential in later phases of infection and has a role in reinfection suppression. Coronavirus-specific antibodies were identified in 80 to 100% of patients with SARS-CoV-1 and MERS two weeks after the disease onset, with delayed immune response in severe infection (42–44). A systematic review in 2020 reported that antibodies to coronavirus were infrequently seen in the first seven days after infection but increased in the second and third weeks of infection (45). ADE (antibody-dependent enhancement) was reported to play a role in previous SARS and MERS infections (6). It is unknown whether antibodies are associated with disease severity.

Due to the similarities in the immune responses between MERS and SARS-CoV-2 infections, particularly the T cell response, several authors hypothesized potential cross-reactive immunity between the two pathogens (46). Yaqinuddin (16) hypothesized that T cells contain specific receptors (TCRs) that may recognize the SARS-CoV-2 peptides in individuals with previous MERS-CoV infection due to sequence homology between the two viruses. In experimental models, variable degrees of cross-reactivity between T- and B-cell antibodies against SARS-CoV-2 and MERS-CoV were detected (19, 47). In the present report, we found that patients with previously confirmed MERS-CoV infection had a lower risk of SARS-CoV-2 infection than the MERS-CoV-negative group. In Barry et al. (18), there was no co-occurrence of MERS-CoV among SARS-CoV-2-infected persons in the KSA. Such findings are crucial in vaccine development against the ongoing COVID-19 pandemic, as understanding the immune response against human CoVs could help in the vaccine development process. According to a large-scale Korean study, serum antibodies against the spike antigen of MERS-CoV persist for three years after infection (48). A more recent report detected MERS-CoV–specific neutralizing antibodies for six years post-infection in 48 patients with previous MERS-CoV infection (20), which is significantly longer than the reported duration for serum antibodies against the spike antigen of SARS-COV-2 (nearly eight months) (49). In the present study, the median duration from MERS-CoV infection and SARS-CoV-2 infection was 3.4 years. We argue for future multicenter immunological studies from MERS-CoV endemic areas to characterize the potential cross-reactivity between MERS-CoV and SARS-COV-2 infections, aiming to guide ongoing research on vaccine development.

The virulence mechanism of SARS-COV-2 is thought to depend on its nonstructural and structural proteins. ACE2 is the receptor of SARS-COV-2 within the body and is predominately expressed in type II alveolar cells (50). Once the virus binds with its receptor, ACE2 expression is markedly elevated, leading to alveolar cell damage and subsequent immune and inflammatory reactions within the body. Previous reports showed that patients with COVID-19 showed pulmonary edema and important proteinaceous exudates, such as large protein globules (51). In severe form, a ‘cytokine storm’ can be initiated by the release of interleukin-6 (IL-6) and other pro-inflammatory cytokines, leading to wide disturbance in thermoregulation, lymphocytes, central nervous system functions, and eventually death (52). Cardiac involvement, such as acute myopericarditis and microthrombi of coronary arteries, was reported in patients with COVID-19 (53). Similar to SARS-CoV-2, MERS-CoV infection is characterized by excessive inflammatory reactions and cytokine release (54). Yaqinuddin (16) hypothesized that cross-reactive immunity can potentially reduce the risk of severe COVID-19 infection in individuals with previous MERS-CoV infection. In contrast, our observations showed that patients with MERS-CoV infection had higher risks of COVID-19-related hospitalization and death than MERS-negative individuals, though the significance was lost when adjusting for age and gender. While the exact mechanisms of severe COVID-19 infection in patients with MERS-CoV infection are unclear, such observations may be explained by the results of previous experiments in MERS-CoV models. Human CoVs can develop immune-escape mechanisms; it was previously noted that MERS-CoV reduces memory T-cell activation through decreased antigen presentation to major histocompatibility complex (MHC) class I and II (55). In addition, MERS-CoV was found to induce T cell apoptosis and severe immunosuppression (56). Thus, patients with previous MERS-CoV may be more susceptible to acquired immune escape mechanisms and more severe immunosuppression. Mutations in the receptor-binding domain (RBD) of the S protein of human CoV were reported to account for the impaired humoral immune response to COVID-19 infection (57, 58). A notable fraction of COVID-19 patients were found to harbor genetic variants in the immune response to infection by human CoV which render them susceptible to severe diseases, as recently reported for defects in type I IFN immunity and the presence of autoantibodies to type I IFN (59, 60). We hypothesize that patients with previous MERS-CoV may carry a higher risk of developing mutations than unexposed individuals. However, our findings should be interpreted cautiously, as MERS-CoV cases in our study were significantly older and had a higher prevalence of comorbidities, which might have represented potential confounders to the study findings. In addition, a previous case report showed a mild COVID-19 infection in a healthcare worker with co-occurrence of MERS-CoV infection (22). As stated above, future multicenter immunological studies from MERS-CoV endemic areas should be conducted to explore the relationship between COVID-19 severity and previous MERS-CoV infection.

The current body of evidence highlights that the risk of SARS-CoV-2 infection and severe disease is closely related to a wide range of patient-specific factors, including age and the presence of comorbidities (61, 62). It was noted that healthcare workers had a higher risk of SARS-CoV-2 infection, and they roughly account for one-quarter of the global COVID-19 cases (63). Healthcare workers had a higher chance of being exposed to patients with SARS-CoV-2 infection; besides, insufficient information about COVID-19 transmission and clinical symptoms can contribute to the higher risk of infection among healthcare workers (64). The present study found that being a healthcare worker and having >1 comorbidity were independent predictors of COVID-19 infection.

The published literature is scarce concerning the potential cross-reactive immunity between SARS-CoV-2 and previous CoVs. To our knowledge, this is the first comparative study that assessed the relationship between previous MERS-CoV and the risk of COVID-19 infection in a MERS-CoV endemic area. However, we acknowledge that the present study had some limitations. The present study data were collected retrospectively with many potential biases, such as case ascertainment bias. Most of the patient variables were self-reported and could not be verified. Besides, the patients’ medical records did not contain information about the MERS-CoV and SARS-CoV2 specific IgG antibodies levels; the measurement of MERS-CoV and SARS-CoV2 specific IgG antibodies are required to elucidate the mechanism underlying the protection. Another limitation is that the sample size calculation of the present study was based on a small pilot study that included 40 participants only. The MERS-CoV cases in our study were significantly older and had a higher prevalence of comorbidities, which might have represented potential confounders to the study findings. In addition, the MERS-CoV positive individuals had a higher percentage of influenza vaccination than the MERS negative group; according to previous reports, influenza vaccination provides bystander immunity to a wide range of viral infections (65). Thus, the history of influenza vaccination might have represented a potential confounder to the study findings.

## Conclusion

In conclusion, individuals with previous MERS-CoV infection can exhibit a cross-reactive immune response to SARS-CoV-2 infection. In the present clinical study, the incidence of SARS-CoV-2 infection was lower in individuals with MERS-CoV infection than in MERS-CoV-negative individuals. This potential cross-reactive immunity can guide ongoing global efforts to develop effective vaccines against the COVID-19 pandemic, particularly with the reported “relatively” long duration of serum neutralizing antibodies against MERS-CoV compared to neutralizing antibodies against SARS-CoV-2. Nonetheless, many gray areas need to be addressed before translating our findings to clinical application. First, the adequacy of the T-cell response elicited by MERS-CoV against SARS-CoV-2 clinical disease should be widely characterized by future research. In addition, long-term studies are recommended to study the longevity of this cross-reactive immunity.

On the other hand, our study demonstrated that patients with MERS-CoV infection had higher risks of COVID-19-related hospitalization and death than MERS-CoV-negative individuals. Experimental research should identify factors that contribute to the dysregulated immune response and immunopathology in patients with severe COVID-19 disease and the co-occurrence of MERS-CoV infection.

## Data Availability Statement

The original contributions presented in the study are included in the article/supplementary material, further inquiries can be directed to the corresponding author.

## Ethics Statement

The studies involving human participants were reviewed and approved by Ministry of Health, Saudi Arabia. Written informed consent for participation was not required for this study in accordance with the national legislation and the institutional requirements.

## Author Contributions

AK contributed to concept and design, definition of intellectual content, literature search, clinical studies, data acquisition, statistical analysis, manuscript preparation, manuscript editing, and manuscript review. AAl contributed to concept and design, literature search, clinical studies, data acquisition, statistical analysis, manuscript preparation, manuscript editing, and manuscript review. YA, FA, YA, and AAl contributed to literature search, clinical studies, statistical analysis, manuscript editing, and manuscript review. SA, MK, SY, GC, AAs, and HJ contributed to literature search, clinical studies, data acquisition, data analysis, statistical analysis, manuscript preparation, manuscript editing, and manuscript review. All authors contributed to the article and approved the submitted version. The requirements for authorship as stated earlier in this document have been met, and each author believes that the manuscript represents honest work.

## Conflict of Interest

The authors declare that the research was conducted in the absence of any commercial or financial relationships that could be construed as a potential conflict of interest.

## Publisher’s Note

All claims expressed in this article are solely those of the authors and do not necessarily represent those of their affiliated organizations, or those of the publisher, the editors and the reviewers. Any product that may be evaluated in this article, or claim that may be made by its manufacturer, is not guaranteed or endorsed by the publisher.
